# Detecting Shared Genetic Architecture Among Multiple Phenotypes by Hierarchical Clustering of Gene-Level Association Statistics

**DOI:** 10.1534/genetics.120.303096

**Published:** 2020-04-03

**Authors:** Melissa R. McGuirl, Samuel Pattillo Smith, Björn Sandstede, Sohini Ramachandran

**Affiliations:** * Division of Applied Mathematics, Brown University, Providence, Rhode Island 02912; † Center for Computational Molecular Biology, Brown University, Providence, Rhode Island 02912; ‡ Department of Ecology and Evolutionary Biology, Brown University, Providence, Rhode Island 02912; § Data Science Initiative, Brown University, Providence, Rhode Island 02912

**Keywords:** GWAS, hierarchical clustering, genomics

## Abstract

Emerging large-scale biobanks pairing genotype data with phenotype data present new opportunities to prioritize shared genetic associations across multiple phenotypes for molecular validation. Past research, by our group and others, has shown gene-level tests of association produce biologically interpretable characterization of the genetic architecture of a given phenotype. Here, we present a new method, Ward clustering to identify Internal Node branch length outliers using Gene Scores (WINGS), for identifying shared genetic architecture among *multiple* phenotypes. The objective of WINGS is to identify groups of phenotypes, or “clusters,” sharing a core set of genes enriched for mutations in cases. We validate WINGS using extensive simulation studies and then combine gene-level association tests with WINGS to identify shared genetic architecture among 81 case-control and seven quantitative phenotypes in 349,468 European-ancestry individuals from the UK Biobank. We identify eight prioritized phenotype clusters and recover multiple published gene-level associations within prioritized clusters.

## Abstract (precis)

McGuirl *et al.* present a new method, Ward clustering to identify Internal Node branch length outliers using Gene Scores (WINGS), for identifying shared genetic architecture among multiple phenotypes. Past research...

SINCE the 2007 publication of the Wellcome Trust Case Control Consortium’s landmark genome-wide association (GWA) study of seven common diseases using 14,000 cases and 3000 common controls, GWA studies have grown dramatically in scope. Much attention has been given to the increasing number of individuals sampled in GWA studies (198 studies to date have analyzed over 100,000 individuals, data accessed at https://www.ebi.ac.uk/gwas/docs/file-downloads on 5 January 2019), as well as to the challenges of interpreting and validating the statistically associated variants identified in large-scale studies (for recent examples, see Hormozdiari *et al.* 2015; Shi *et al.* 2016; Boyle *et al.* 2017; Evangelou *et al.* 2018; Feldman and Ramachandran 2018; Huffman 2018; Zhang *et al.* 2018). However, as “mega-biobank” datasets [used here as by Huffman (2018) to mean “a study with phenotype and genotype data on >100,000 individuals rather than to the physical sample repository”] such as the UK Biobank (Bycroft *et al.* 2017 *preprint*) and BioVU at Vanderbilt University (Roden *et al.* 2008; Denny *et al.* 2010) are interrogated by medical and population geneticists, there are new opportunities to develop approaches for analyzing multiple phenotypes in a single genomic study.

In particular, a fundamental question mega-biobanks can answer is whether shared genetic architecture among multiple phenotypes is detectable using summaries of germline genetic variation. Pickrell *et al.* (2016) explicitly tested for pleiotropy among 42 complex traits, focusing on identifying colocalized variants in GWA studies for pairs of traits [see also Hormozdiari *et al.* (2016), who tested for colocalization between eQTLs and associated variants for the same phenotype]. While phenome-wide association studies (PheWAS; Denny *et al.* 2013, 2016) and multivariate GWA studies have tested for statistical association between variants and multiple phenotypes (Jiang and Zeng 1995; Marchini *et al.* 2007; Ferreira and Purcell 2008; Stephens 2013; Turley *et al.* 2018), these studies (including Hormozdiari *et al.* 2016; Pickrell *et al.* 2016) share the central challenge of single-phenotype GWA studies: they gain power by focusing on variant-level associations but are difficult to interpret biologically. Variant-level association tests further ignore potential genetic heterogeneity across cases, which can manifest on multiple levels: for a given phenotype, cases may harbor different variants in the same causal gene, or the same pathway may be mutated in multiple ways to produce the same phenotype (Leiserson *et al.* 2013).

As large-scale GWA studies find statistically associated variants spread uniformly throughout the genome (Visscher *et al.* 2006; Shi *et al.* 2016; Boyle *et al.* 2017) and that effect sizes have reached diminishing returns (Zhang *et al.* 2018), gene-level association tests (Liu *et al.* 2010; Wu *et al.* 2011; Lamparter *et al.* 2016) can offer insight into gene sets and pathways that are enriched for mutations in cases of a phenotype of interest. Gene-level association tests not only allow for different mutations to be associated with the phenotype of interest in different cases but also generate biologically interpretable hypotheses regarding genetic interactions that the GWA framework ignores (Zuk *et al.* 2012). Despite this, gene-level association tests have rarely been brought to bear on multivariate GWA datasets. One approach was developed by Chang and Keinan (disPCA; Chang and Keinan 2014), who applied principal components analysis (PCA) to a matrix of gene-level association scores to detect clusters of phenotypes in two dimensions. However, their dimensionality reduction of the gene score matrix ignored minor axes of variation across gene scores for ease of visualization, and distances between phenotypes in principal components (PC) space were difficult to interpret. Thus, identifying phenotypes significantly enriched for shared mutations in mega-biobanks remains an open challenge.

In this study, we present Ward clustering to identify Internal Node branch length outliers using Gene Scores (WINGS), a flexible method for (*i*) computationally detecting phenotype clusters based on gene-level association scores, and (*ii*) ranking phenotype clusters based on their levels of significance. Given gene-level association test statistics for multiple phenotypes as input, WINGS enables the detection of a “core set” of genes—that is, genes enriched for mutations in cases—across multiple phenotypes. WINGS allows for the identification of potentially pleiotropic genes (genes that play a role in the development of multiple phenotypes). We hypothesize that genes with a shared significance across phenotypes will drive the formation of biologically distinct clusters. For a given cluster of phenotypes, those genes that are significant should be considered primary candidates for drug design. In this study, we are particularly interested in the ability to detect shared significant genetic architectures among phenotypes using only common variants.

To identify genetic architectures shared across a set of phenotypes, we aggregate SNP-level association statistics using PEGASUS (Nakka *et al.* 2016, 2017). PEGASUS can calculate a region-level association *P*-value for any set of the user-defined genomic region or compute gene-level association statistics. Here, we choose the latter since our ultimate goal is to identify core genes enriched for mutations in cases across multiple phenotypes. To each phenotype, PEGASUS assigns a feature vector (see section *Overview of WINGS pipeline* for more details) whose elements are gene-level association *P*-values scores, or “gene scores.” Each feature vector of gene scores is an element of a high-dimensional vector space whose dimension is given by the number of genes included in the GWA study data. Given a list of *N* phenotypes, this approach therefore yields *N* feature vectors. The more significant genes two phenotypes share, the closer their features vectors will be. Choosing a norm on the vector space in which the feature vectors lie allows us to compute pairwise distances between any two feature vectors, resulting in an *N* × *N* matrix of pairwise distances—we note that different norms will result in different distance matrices, and we use this fact in this study to emphasize different parts of a feature vector when identifying prioritized clusters (herein, “prioritized clusters” refers to groups of phenotypes with very strong affinity). Once a distance matrix has been computed, we can use clustering algorithms (in our case, Ward hierarchical clustering) to divide the set of phenotypes into disjoint groups that separate feature vectors based on their pairwise distances.

While hierarchical clustering algorithms have proven effective across a range of applications (Aceto *et al.* 2014; Brown *et al.* 2014; Pagnuco *et al.* 2017), the typical output of these clustering methods is a dendrogram illustrating the sequential formation of clusters starting with each cluster containing only a single data point and ending with a single cluster containing all of the data points. Consequently, it is unclear how to distinguish prioritized clusters from ordinary clusters, and often this is done by choosing a single cutoff height in the dendrogram or predetermining the number of desired clusters (Langfelder *et al.* 2008; Hastie *et al.* 2009; Zambelli 2016). WINGS, by contrast, implements a multi-step algorithm to systematically identify and rank prioritized clusters, described in detail in *Materials and Methods*. We evaluate the performance of WINGS in simulations under a variety of genetic architectures within phenotypes and shared among phenotypes. Lastly, we apply WINGS to identify prioritized phenotype clusters across 81 case-control phenotypes and seven quantitative phenotypes assayed in 349,468 unrelated European-ancestry individuals in the UK Biobank.

## Materials and Methods

Following initial quality control (QC steps) (see Supplemental Material, Section S1), 349,468 individuals who self-identified as British and 410,172 variants remained for analysis. In order to control for population structure within the remaining cohort, PCA was performed using flashpca (version 2.0; Abraham *et al.* 2017) on SNPs passing QC that were also in linkage equilibrium (SNPs with *r*^2^ > 0.1 removed, resulting in 104,834 SNPs for PCA).

We analyzed phenotypes in two stages. We selected an initial set of 26 case-control phenotypes based on phenotypes (see Supplemental Material, Table S1) that had been previously analyzed in Shi *et al.* (2016) and Pickrell *et al.* (2016) that also had at least 100 cases in our cohort. Those phenotypes that did not have at least 100 cases in our cohort after QC were not included in the analysis. A GWA study was performed for each of these 26 case-control phenotypes using plink2 (Chang *et al.* 2015) including age, sex, and the first 10 principal components as covariates to control for population structure.

We then expanded our analysis to include 55 additional case-control phenotypes and seven quantitative phenotypes from the UK Biobank (see Table S1). These phenotypes were selected only if they had >1000 cases in the analyzed cohort and had positive heritability estimates calculated using linkage disequilibrium (LD) score regression (Bulik-Sullivan *et al.* 2015b).

### Overview of WINGS pipeline

For each of the phenotypes being jointly studied (either in simulations, as detailed in the next subsection, or in the UK Biobank), we used PEGASUS (Nakka *et al.* 2016) to calculate gene-level association *P*-values for all autosomal genes in the human genome with at least one SNP within a ±50 kb window (17,651 genes). PEGASUS, developed by our group (Nakka *et al.* 2016, 2017), models correlation among genotypes in a region using LD, the same model as VEGAS (Liu *et al.* 2010) and SKAT without weighting rare variants (Wu *et al.* 2011). PEGASUS, by contrast, achieves up to machine precision in gene-level association statistic computations via numerical integration. In this study, we refer to the −log_10_ transformed PEGASUS gene-level association statistics as “gene-scores.”

We then concatenated together each phenotype’s feature vector to generate a phenotype by gene matrix, the ultimate input for the WINGS software. Next, Ward hierarchical clustering (Ward 1963; Ward and Hook 1963) was applied to the phenotypes using the PEGASUS gene scores (−log_10_ transformed PEGASUS *P*-values) as feature vectors.

**Figure 1:**
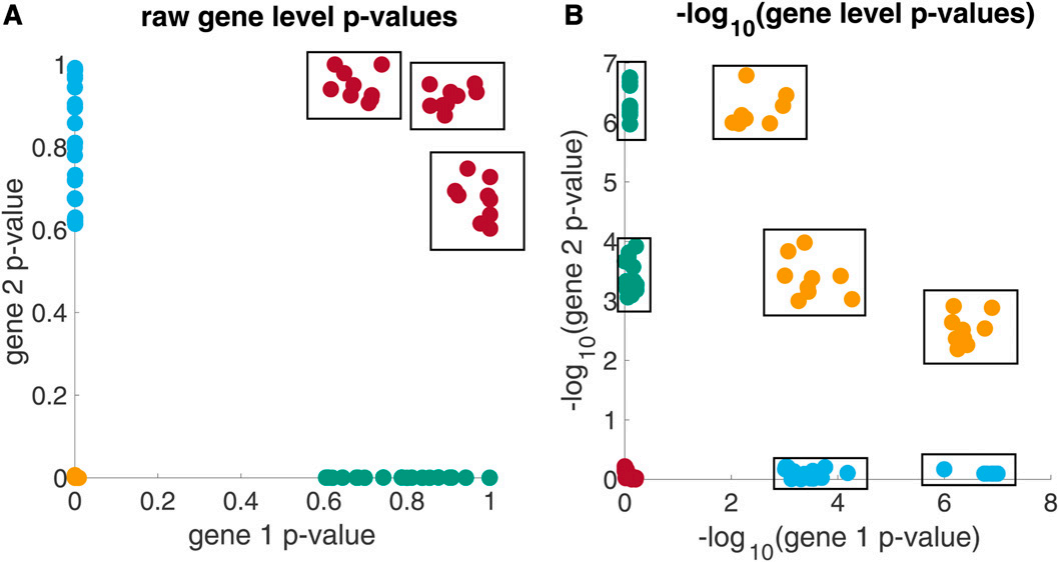
Synthetic clusters of phenotypes with (A) shared nonsignificant genetic architecture and (B) shared significant genetic architecture from the raw and –log_10_ scales, respectively. Schematic example showing (A) simulated 2-dimensional gene-level *P*-values and (B) their corresponding −log_10_ transformed gene-level *P*-values. The boxed groups of points represent clusters of shared nonsignificant genetic architecture (A) and clusters of shared significant genetic architecture (B).

The primary motivation for using the −log_10_ transformation is that, for a given dimension (*i.e.*, a gene), it emphasizes the phenotypes for which *P*-values are significant (see Figure 1). Biologically, this transformation can be interpreted as a rescaling of *P*-values that emphasizes, for a given gene, mutations enriched in multiple phenotypes. Moreover, by using the −log_10_ transformation together with a distance metric, we can measure the similarity between gene scores for each phenotype and perform our clustering analysis. While genetic correlations could also be used as inputs for some clustering analyses, correlations do not satisfy the triangle inequality, making resulting clusters difficult to interpret. For example, using genetic correlations, phenotype A and phenotype B, as well as phenotype B and phenotype C could be highly correlated, even though phenotype A and phenotype C are highly uncorrelated: it is unclear how phenotypes A, B, and C would and should cluster together in this framework.

In our analyses of the UK Biobank, a set of seven continuous phenotypes were clustered separately due to their comparatively much larger sample sizes (Figure S1 shows how the continuous and binary phenotypes cluster when treated as a single data set). Prioritized clusters were identified and ranked using the WINGS branch length thresholding algorithm (described in the next section).

### WINGS, a new method for automatic phenotype cluster detection and ranking

WINGS is a thresholded hierarchical clustering algorithm that takes a matrix of gene-level association test results as its input and outputs identified phenotype clusters ranked by their significance. First, WINGS applies Ward hierarchical clustering to the matrix of gene-level association test results, which we compute using PEGASUS. Specifically, consider a data set with *N* data points. Ward hierarchical clustering is an agglomerative clustering algorithm: initially, there are *N* clusters, each containing exactly one data point, and clusters are merged recursively in a hierarchical manner until there is a single cluster containing all *N* data points (Ward 1963; Ward and Hook 1963; Hastie *et al.* 2009).

Using an objective function approach, at each stage in an agglomerative clustering algorithm, the pair of clusters that minimizes *the merging cost* are combined to form a single cluster. For Ward hierarchical clustering, the merging cost for combining clusters R and S of size *N_R_* and *N_S_*, respectively, is defined as

$$d\left(R,S\right)=\sqrt{\frac{N_{R}\cdot N_{S}}{N_{R}+N_{S}}}{\left\|C_{R}-C_{S}\right\|}_{2},$$

where *C_R_* and *C_S_* are the centroids of clusters R and S, respectively, and ‖·‖_2_ denotes the Euclidean norm. Note, this merging cost is equivalent to minimizing the increased sum of squared errors (Ward 1963; Ward and Hook 1963; Hastie *et al.* 2009).

The choice to use Ward as the linkage criteria for WINGS was not arbitrary. Ward hierarchical clustering focuses on minimizing differences within the clusters, rather than maximizing pairwise distances between clusters. Previous work on comparing different agglomerative hierarchical clustering algorithms suggests that Ward clustering performs best when clustering high dimensional, noisy data as long as cluster sizes are assumed to be approximately equal (Ferreira and Hitchcock 2009; Hossen *et al.* 2015). We also note that we applied other linkage criteria to the data for comparison (see Supplementary material Section S3 and Figures S2–S7 for more details).

Hierarchical clustering results are often represented in a dendrogram, where each branch corresponds to a cluster, but it is not clear how to extract the prioritized clusters, or clusters with the strongest affinity (Langfelder *et al.* 2008; Hastie *et al.* 2009; Zambelli 2016). Intuitively, prioritized clusters are those that form early on in the hierarchical clustering algorithm, and do not merge with other clusters until there are very few clusters left. This corresponds to clusters that form near the bottom of the representative dendrogram tree and have long branch lengths.

To quantitatively define the notion of sufficiently long branch length, we look at the consecutive differences between branch lengths and search for large gaps in the branch length distributions. That is, in the second step of WINGS, we implement the following branch length thresholding algorithm to identify prioritized phenotype clusters within a dendrogram:

1. Sort all the branch lengths corresponding to small clusters (small clusters are those containing *T* phenotypes or fewer, where *T* is the user-defined cluster size threshold);
2. Calculate the consecutive differences between branch lengths to get the branch length gaps;
3. Identify branch length gaps that are more than three scaled median absolute deviations away from the median, and classify these as branch length gap outliers;
4. Set the branch length threshold to be the minimum branch length such that the branch length is greater than the median of all branch lengths and its branch length gap is a branch length gap outlier. If this threshold does not exist, we conclude that there are no prioritized clusters.

Finally, prioritized clusters are identified as the clusters whose corresponding dendrogram branch length is greater than, or equal to, the branch length threshold defined above. Pseudocode for WINGS can be found in Algorithm 3, along with pseudocode for the Ward hierarchical clustering algorithm and branch length thresholding algorithm in Algorithms 1–2.

The branch length thresholding algorithm in WINGS is a multi-step process for identifying prioritized clusters in a dendrogram that does not require prior knowledge of the number of desirable clusters and is more flexible than the traditional fixed branch cut methods (Zambelli 2016). Previous work in Langfelder *et al.* (2008) similarly introduces a dynamic method for identifying clusters from a dendrogram tree. In contrast to the iterative tree-cut algorithms presented in Langfelder *et al.* (2008), however, WINGS relies solely on the dendrogram branch lengths and does not rely on making any tree cuts.

Notably, there are only two parameter choices in WINGS: the cluster size threshold and the outlier criterion. The cluster size threshold is a user-defined parameter that controls the scale of the prioritized clusters. As the cluster size threshold decreases, WINGS will prioritize smaller clusters of phenotypes that share a higher percentage of genes enriched for mutations, in comparison to supersets of phenotypes containing the prioritized clusters. In our experiments, we use a cluster size threshold of eight, and the default cluster size threshold in the WINGS software is conservatively set to $\left\lceil \frac{N}{3}\right\rceil$.

The second WINGS parameter—the outlier criterion for branch length gaps—is set to three scaled median absolute deviations away from the median. This median absolute deviation method for identifying outliers is easy to compute, robust, and not dependent on sample size (in our application, number of phenotypes) (Rousseeuw and Croux 1993; Huber 2011; Leys *et al.* 2013; MATLAB Data Import and Analysis 2018); moreover, the choice of using an outlier threshold of three scaled median absolute deviations away from the median is appropriately conservative as our data are high-dimensional (Leys *et al.* 2013).
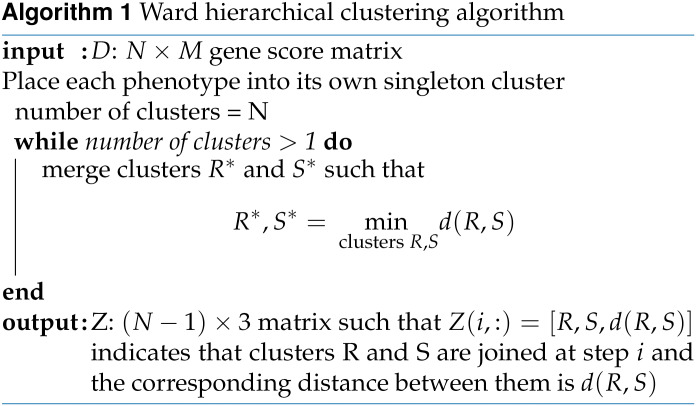

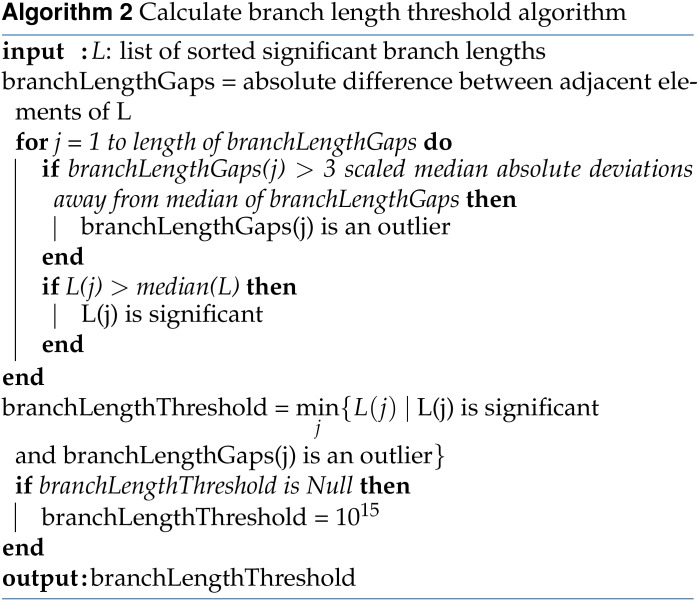

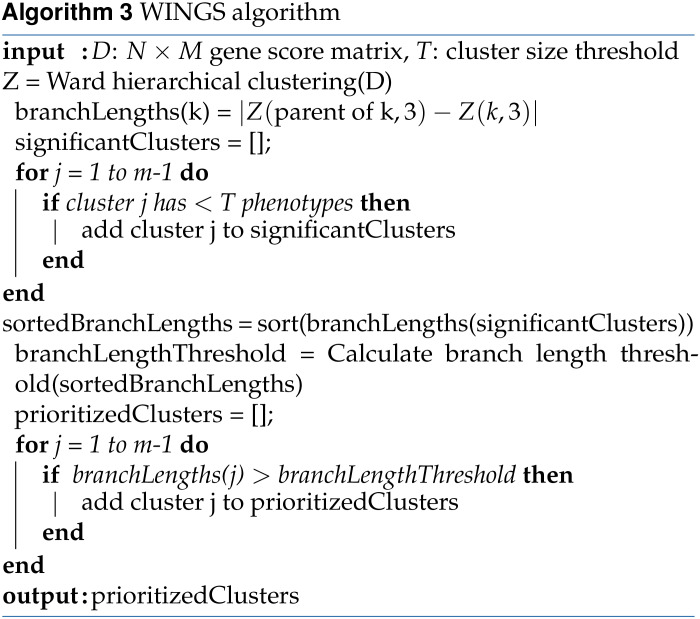


In the section *Parameter sensitivity analysis*, we test the sensitivity of WINGS with respect to both the cluster size threshold and the outlier criterion. These results provide intuition for how to choose the user-defined parameter values, as well as demonstrate the robustness of WINGS to the choice of these parameters. WINGS was implemented in MATLAB (R2017b) and applied to both simulated gene score matrices and empirical PEGASUS gene scores for phenotypes in the UK Biobank. These results are presented in *Results*.

### Simulations of phenotypes with shared genetic architecture

To test the sensitivity of WINGS when identifying both ground truth shared genetic architecture and varying levels of random noise in gene-level association *P*-values, we first applied WINGS to simulated gene score matrices. To accomplish this task, we generated “shared significant genetic architectures,” where shared genes have a PEGASUS gene-level *P*-value < 2.83 × 10^−6^.

Gene scores obtained as −log_10_ transformed PEGASUS gene-level *P*-values range from (0, ∞), where highly significant genes have high transformed gene scores. We expect that clusters in this space are driven by shared significant genetic architecture—that is, phenotypes that have a high percentage of shared significant genes—since these features contribute the most to the pairwise distances between phenotypes. If, instead, we studied the raw (untransformed) PEGASUS gene-level *P*-values, we expect to see clusters of shared nonsignificant genetic architecture, referring to phenotypes that have a high percentage of shared nonsignificant genes.

This distinction is illustrated in the synthetic example shown in Figure 1. As shown in Figure 1A, groups of shared nonsignificant genetic architecture (in red) form clusters on the raw scale, whereas phenotypes with shared significant genetic architecture (in orange) reside as a large, and therefore nonprioritized, group in the bottom left-hand corner of the plot. In contrast, in Figure 1B, groups of shared significant genetic architecture form clusters on the −log_10_ scale since this transformation maps the small region of significant *P*-values (gene-level *P*-value $<2.83\times 10^{-6}$) to the much larger region of (3, ∞).

The WINGS thresholding algorithm prioritizes phenotype clusters based on shared significant gene-level associations. As detailed in our simulation protocol below, our simulation design preserves shared significant genetic architecture, and we allow the rest of the gene score matrix to result from a random draw to simulate both shared genetic architecture and surrounding noise among phenotypes. While genes in physical proximity and genes that interact will likely have correlated gene scores, we did not specifically simulate such local correlation among gene scores (but see Figures S10 and S20).

Each simulated matrix was generated by randomly selecting PEGASUS *P*-values from the empirical distribution of PEGASUS *P*-values for Crohn’s disease (ICD10 code K50; 1453 cases, 348,015 controls among the cohort passing our QC steps detailed in Supplementary Section S1). PEGASUS *P*-values were then partitioned into significant (*P*-value $<2.83\times 10^{-6}$) and nonsignificant (*P*-value $\geq 2.83\times 10^{-6}$) groups (Wojcik *et al.* 2015). In the protocol described below, scores were taken randomly from the empirical gene scores in each of these groups. All simulated matrices maintain the same number of features (17,651 PEGASUS gene-level *P*-values, one for each autosomal gene) as our empirical analyses. For each phenotype in the matrix, 1% (175) of genes were assigned a significant value (*P*-value $<2.83\times 10^{-6}$).

We designed simulations that varied along two major parameters. We first set the number of phenotypes analyzed to either 25, 50, 75, or 100. Second, we set the percentage of the 175 significant genes that are shared between all cluster phenotypes to either 1% (2 genes), 10% (18 genes), 25% (44 genes), 50% (88 genes), or 75% (131 genes) as shared genetic architecture. For every pair of the parameters above, we performed 1000 simulations as detailed below.

In every simulation, the number and size of the clusters were determined using the following protocol:

1. Choose M from a uniform distribution between 3% and 15% of the total number of phenotypes; M will be the number of ground truth clusters simulated (*e.g.*, for simulations with 100 phenotypes they all contain between three and 15 clusters).
2. For j=1,2,…,M:
  a. Generate ground truth cluster *j* of randomly selected phenotypes whose size is drawn at random from a uniform distribution between two and eight;
  b. Select the corresponding percentage of significant genes to be shared for all phenotypes in the ground truth cluster;
  c. Remove phenotypes in ground truth cluster *j* and corresponding shared significant genes from their respective pools (a phenotype may only be in one ground truth cluster, and a gene can only be shared and significant in one ground truth cluster); and
  d. Assign nonshared significant genes and nonsignificant genes to each phenotype in the ground truth cluster
3. For all phenotypes not assigned to a ground truth cluster in Step 2, randomly draw 175 genes that remain in the pool to be significant, and assign remaining genes as nonsignificant.

### Architecture of shuffled gene score matrix for 81 phenotypes

Some of the phenotypes being studied here are highly polygenic, and, consequently, overlapping genetic architecture might occur due to inherent randomness in the data (Jordan *et al.* 2019 *preprint*). We performed a permutation test to assess whether the prioritized clusters identified by WINGS are connected based on significant overlapping genetic architectures rather than random correlations. Specifically, we shuffled each phenotype’s gene scores. We then applied WINGS to the shuffled matrix to understand if prioritized clusters formed by chance. We found that across 1000 random shuffles WINGS identified, on average (with small SD), five prioritized clusters of size two and one prioritized cluster of size three, and these clusters do not overlap with the prioritized clusters from the empirical data. WINGS did not identify any prioritized clusters of size four or more, and the average number of prioritized clusters approaches zero (with small SD) as cluster size increases for the randomly shuffled data (Figure S10).

When applying WINGS to the −log_10_ empirical gene-score matrix, there are seven prioritized clusters consisting of four or more phenotypes, four clusters of three phenotypes, and seven clusters of size two; moreover, three of the seven two-phenotype clusters and three of the four three-phenotype are subclusters of larger prioritized clusters (Table 3). The size of the prioritized clusters from the empirical data are consistently greater than the average number across the randomly shuffled data, providing evidence that the empirical prioritized clusters are not linked by chance. Lastly, we note that the dendrograms from these randomly shuffled simulations (not shown) are flat in comparison to the empirical dendrogram, Figure 3. A flat hierarchical clustering structure indicates homogeneous data as expected from random data, providing further evidence that the empirical prioritized clusters are formed from having a significantly higher degree of overlapping genetic architecture and not due to random correlations.

### Comparison of WINGS to cross-trait LD score regression, disPCA, and other regional trees

To illustrate the power of WINGS to prioritize clusters of phenotypes with shared genetic architecture, we compared output from WINGS with that of cross-trait LD score regression analysis (Bulik-Sullivan *et al.* 2015b). For each pair of 81 phenotypes we analyzed, we calculated the cross-trait coheritability using variant level GWA summary statistics as described in Bulik-Sullivan *et al.* (2015b). For each prioritized cluster identified by WINGS, we then determined what proportion of phenotype pairs had a significant correlation according to cross-trait LD score regression (Table 4). Cross-trait LD score regression uses variant level summary statistics to estimate the genetic coheritability of two traits (Bulik-Sullivan *et al.* 2015a). After correcting for inflation between variant summary statistics induced by LD, traits with coordinated statistical signals across loci are labeled as having significant cross-trait heritability estimates.

Next, for additional performance comparison, we applied WINGS to the first two PCs of a matrix of gene-scores that was created using the minSNP approach (Fehringer *et al.* 2012), which assigns the minimum variant *P*-value within a gene as the gene-level *P*-value (see disPCA methods; Chang and Keinan 2014). The results of this analysis are presented in section *Previous methods*, *and comparative dendrogram analysis* and Figure 4 and Figure S13.

To better understand potential pitfalls in using genes and a ±50 kb boundary as our feature set in our primary analysis, we conducted four additional analyses. First, we included all intergenic regions as features and calculated a PEGASUS regional score for each intergenic region. Second, we performed an analysis that included imputed variants with an imputation score >0.8, while additionally filtering out variants that were in high LD with one another $r^{2}>0.9$ (using plink’s-indep-pairwise 100 10 0.9 flag) in order to reduce the runtime of our analysis. Third, to determine the effect of mapping noncoding variants to nearby genes using the ±50 kb buffer region, we performed an analysis of 17,651 genes with the buffer region set to zero. By setting the buffer region to zero, we omitted any SNPs that were not in the exons or introns of a gene. Finally, to understand if the correlation in gene scores between physically colocated genes was inducing spurious clusters, we parsed the genome into 33,685 independent haplotype blocks using plink’s -blocks command. The details of these four additional feature sets are presented in Table 1, and the results of these analyses are presented in the section *Previous methods and comparative dendrogram analysis*.

**Table 1:** Feature sets for comparative dendrogram analysis of WINGS

Feature set	No. of genes	Intergenic regions included	Results
Intergenic regions*^a^*	20,611	Yes	Figures S14–S15
Imputed genotype data*^b^*	17,678	No	Figures S16–S17
Genes with no buffer region*^c^*	13,031	No	Figures S18–S19
Gene scores per haplotype block*^d^*	33,685	No	Figures S20–S21

Summary of features sets used in our comparative dendrogram analysis of WINGS (see sections *Comparison of WINGS to cross-trait LD score regression*, *disPCA*, *and other regional trees* and *Previous methods and comparative dendrogram analysis*). We present the number of genes in each data set and whether or not the data contain intergenic regions, along with references to corresponding figures that show the results of applying WINGS to each feature set.

a For the intergenic regions set, we included all intergenic regions as features and calculated a PEGASUS regional score for each intergenic region.

b For the imputed data, imputed variants with an imputation score >0.8 were included, and variants that were in high LD with one another $\left(r^{2}>0.9\right)$ were filtered out (using plink’s -indep-pairwise 100 10 0.9 flag) to reduce runtime.

c The feature set with the buffer region set to zero omits any SNPs that were not in the exons or introns of a gene.

d The gene scores per haplotype block set were generated by parsing the genome into 33,685 independent haplotype blocks using plink’s -blocks command.

### Data availability

The authors state that all data necessary for confirming the conclusions presented in the article are represented fully within the article. Shared significant gene lists and results from gene-set enrichment analysis for each of the prioritized clusters in Figure 3, as well as scripts that were used to generate the simulated matrices and implement WINGS, are available at https://github.com/ramachandran-lab/Pegasus-WINGS/. Supplemental material available at figshare: https://doi.org/10.25386/genetics.11781954

## Results

### Performance on simulated data

In Table 2, we report power as the percentage of ground truth simulated clusters that WINGS correctly labels as prioritized across the 1000 simulations, for a fixed number of phenotypes in analysis and percent shared significantly mutated genes (“shared genetic architecture”). We define shared genetic architecture for a cluster to be the percentage of genes that are significant (*P*-value $<2.83\times 10^{-6}$) across all member phenotypes of the cluster. We also measure the precision of WINGS in identifying simulated clusters. We define precision for a given simulation as the number of ground truth clusters that were correctly identified as prioritized, and that further fell within the top *x* prioritized clusters in that simulation. For example, if a simulation has five ground truth clusters, the power of WINGS for that simulation would be the percentage of those five clusters that are identified as prioritized. The precision of WINGS is the percentage of those five ground truth clusters that have been both correctly identified as prioritized and are within the five most prioritized clusters identified in that simulations.

**Table 2:** Power, precision, F1 scores of WINGS across a range of phenotypes included as well as shared genetic architecture

	Shared genetic architecture											
	Power				Precision				F1 score			
N*^a^*	0.1	0.25	0.5	0.75	0.1	0.25	0.5	0.75	0.1	0.25	0.5	0.75
25	97.43	100	100	100	65.77	69.36	71.33	75.39	0.920	0.938	0.936	0.935
50	96.63	100	100	100	52.52	58.76	62.05	63.80	0.818	0.837	0.826	0.821
75	96.81	100	100	100	43.56	52.28	55.04	55.04	0.727	0.767	0.747	0.748
100	96.45	100	100	100	36.39	46.76	50.92	50.92	0.668	0.694	0.690	0.683

Shared genetic architecture denotes the percentage of the 175 significant genes in each phenotype that are shared across all phenotypes in a cluster. Every entry in the table represents 1000 simulations under the corresponding parameters. The power of WINGS for identifying ground truth clusters in simulations is defined as the percentage of ground truth clusters across these 1000 simulations that were identified as prioritized by WINGS. The precision of WINGS is defined as follows: in a simulation with *x* ground truth clusters and a given number of phenotypes and proportion of shared genetic architecture, precision is the percentage of ground truth clusters that were identified as prioritized and within the *x* most prioritized clusters ranked by the branch length thresholding step in WINGS. F1 score is twice the product of precision and recall divided by the sum of precision and recall; in this context, recall is the percentage of ground truth clusters prioritized by WINGS.

a N is the number of phenotypes in the simulation.

Table 2 reports the precision of WINGS on the simulations across varying parameter values for both the number of phenotypes analyzed and shared genetic architecture using PEGASUS *P*-values. We additionally generated simulations using the same protocol but substituting the PASCAL (“max” model) (Lamparter *et al.* 2016) gene-level association test results for PEGASUS gene-level association *P*-values to illustrate that WINGS can be used with any gene-level association metric. The results for the simulations using PASCAL (“max” model) are shown in Table S3. We note the “sum” model of PASCAL is identical to the model of PEGASUS, and so we do not compare WINGS results using these two models for gene scores.

**Figure 2:**
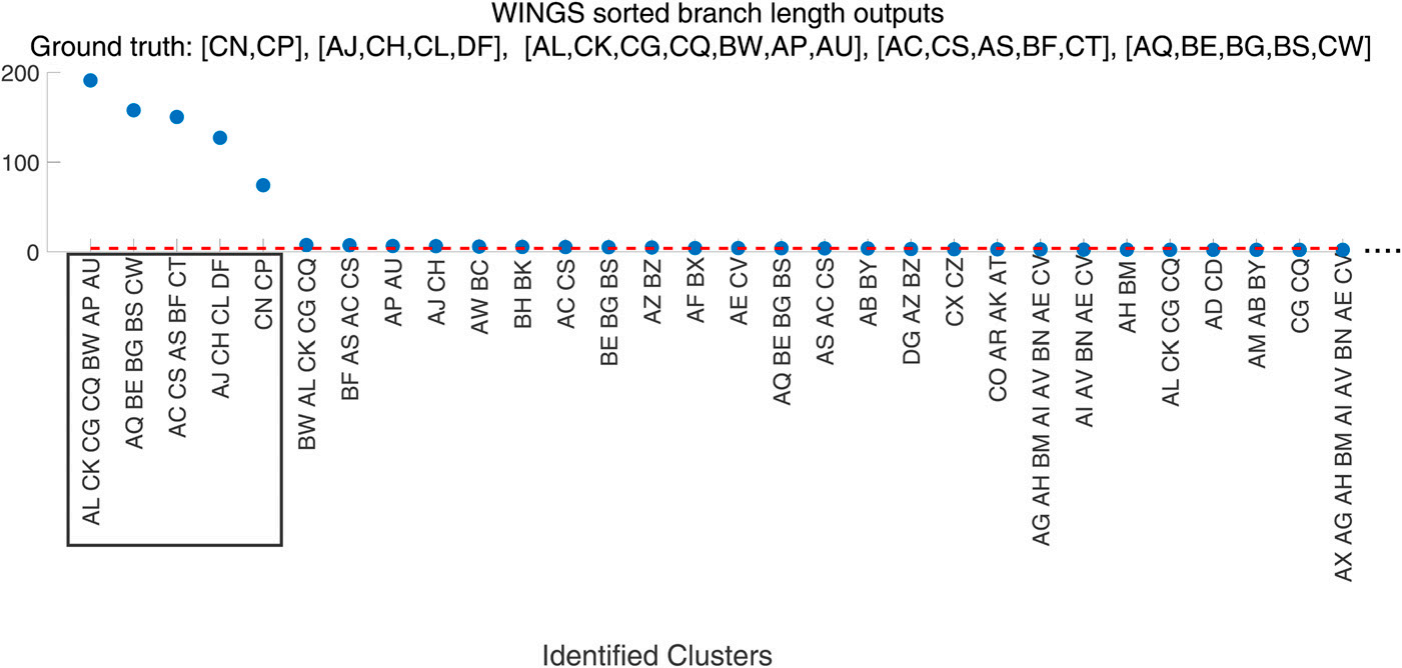
WINGS sorted branch lengths from a simulation identifies prioritized clusters on the −log_10_ scale. We show the sorted branch lengths corresponding to the dendrogram branches generated by WINGS when applied to the −log_10_ transformed PEGASUS gene scores from a simulation with 75 phenotypes, 75% (131) shared genes. For this simulation the ground truth clusters are [CN, CP], [AJ, CH, CL, DF], [AL, CK, CG, CQ, BW, AP, AU], [AC, CS, AS, BF, CT], and [AQ, BE, BG, BS, CW]. The dashed red horizontal line corresponds to the branch length threshold, where the identified prioritized clusters are those lying above the dashed line. The ground truth clusters are correctly identified as the prioritized clusters (boxed). This figure has been truncated on the right (removing some clusters that are not identified as prioritized) for better visualization purposes. The corresponding dendrogram is shown in Figure S12.

One sample output of WINGS applied to a standard simulation is presented in Figure 2, and the corresponding dendrogram is shown in Figure S12. The thresholded hierarchical clustering algorithm within WINGS identifies the ground truth clusters as the top five most prioritized clusters. These results suggest that WINGS applied to −log_10_-transformed gene-level association statistics accurately captures groups of phenotypes that have a high percentage of shared significant genes.

### Analysis of 81 case-control phenotypes

**Figure 3:**
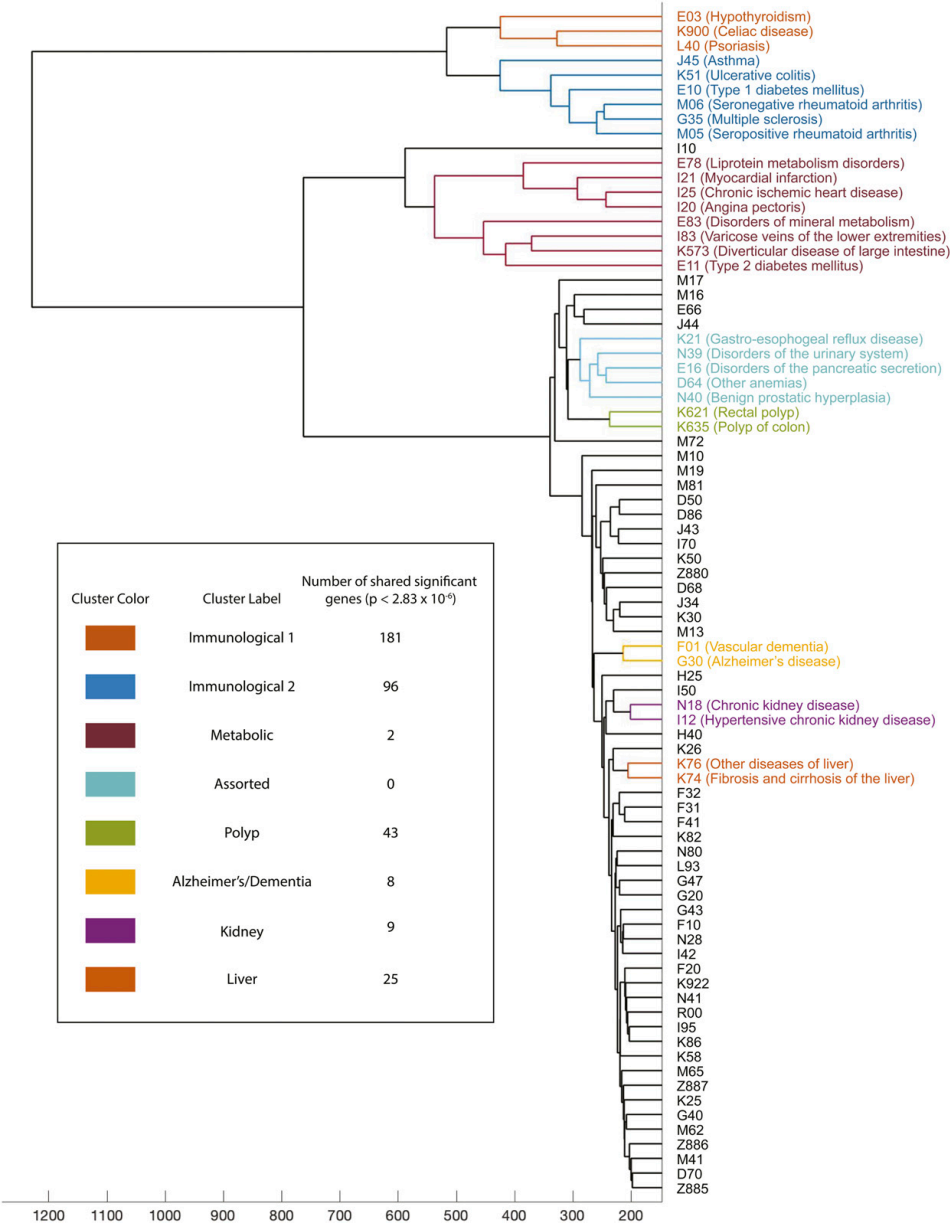
WINGS dendrogram from 81 case-control phenotypes in the UK Biobank reveals clusters of phenotypes with shared significant genetic architecture. We show the dendrogram output from the WINGS analysis of −log_10_ transformed PEGASUS scores of 81 case–control phenotypes in the UK Biobank. Listed are the ICD10 codes and common names of each phenotype that belongs to a prioritized cluster, grouped by cluster. Table insert: Each prioritized cluster’s color, assigned label, and number of shared significant genes (the number of PEGASUS gene scores with *P*-value $<2.83\times 10^{-6}$, Bonferroni corrected for 17,651 autosomal genes). Figure S11 demonstrates how the WINGS algorithm identifies prioritized and nonprioritized clusters using branch lengths from this dendrogram. We suggest viewing this figure alongside Figure S11, as the prioritized clusters may appear arbitrary or nonintuitive to the human eye. We additionally note that a similar analysis using imputed data from the UK Biobank shows similar results (Figure S16) suggesting that shared genetic architectures can be detected using only common genotyped variants.

We first applied WINGS to the 26 case-control phenotypes analyzed in Pickrell *et al.* (2016) and Shi *et al.* (2016). We provide the results of our analysis of these 26 phenotypes in Figures S8 and S9 (Section S4) and discuss our findings in *Discussion*. The focus of this paper is on the application of WINGS to 81 case-control phenotypes from the UK Biobank. We use the 26 phenotypes from our initial analysis and add 55 case-control phenotypes that had at least 1000 cases in our cohort from the UK Biobank (see Supplementary Material, Section S1 for QC details). The additional 55 phenotypes and their corresponding case numbers are provided in Table S1. We then applied WINGS to the resulting 81 phenotypes by 17,651 genes matrix. In this expanded set of phenotypes, WINGS identifies eight prioritized clusters, some of which contain smaller subclusters of phenotypes that are also identified as prioritized clusters. For instance, in Figure 3, the Metabolic contains eight phenotypes, but many of the individual phenotype clades within it are additionally prioritized, including Angina pectoris (I20) and Chronic ischemic heart disease (I25). For an exhaustive list of prioritized subclusters, see Table 3. The eight prioritized clusters, as well as their phenotypes, are shown in the WINGS dendrogram in Figure 3 with the corresponding sorted branch length plots presented Figure S11. Note that we set the cluster size threshold to eight in our empirical analyses.

**Table 3:** Complete list of the −log_10_ prioritized clusters of phenotypes in the analysis of 81 case-control phenotypes

Cluster classification	Phenotypes in cluster
Kidney cluster	I12, N18
Liver cluster	K74, K76
Polyp cluster	K621, K635
Metabolic cluster	I20, I25
	I20, I21, I25
	E78, I20, I21, I25
	I83, K573
	E11, I83, K573
	E11, E83, I83, K573
	E11, E78, E83, I20, I21, I25, I83, K573
Immunological 2 cluster	G35, M05, M06
	E10, G35, M05, M06
	E10, G35, K51, M05, M06
	E10, G35, J45, K51, M05, M06
Assorted cluster	D64, E16, K21, N39, N40
Immunological 1 cluster	K900, L40
	E03, K900, L40
Alzheimer’s/dementia cluster	F01, G30

Phenotypes are denoted here using ICD10 codes; see Table S1 for detailed phenotype names. Clusters that have prioritized subclusters are listed in the hierarchical order in which they merge to form new, larger prioritized clusters (vertically, from top to bottom). See Figure 3 and Figure S11 for the corresponding dendrogram and branch lengths plot.

We find that the case number of a phenotype is not significantly correlated with that phenotype being in a prioritized cluster (Kendall’s *τ*, *P*-value = 0.2625). We additionally compared the vector of gene-level association statistics calculated using only genotyped variants to the vector of gene-level association statistics calculated using the genotyped and imputed variants for each phenotype. The correlation coefficient (r) for each of these comparisons was >0.75, and all were statistically significant (*P* = 0). Finally, we asked how many genes that had previously contained no significant (*P*-value $<5.7\times 10^{-8}$) variant level associations contained at least one such variant after the addition of the imputed data. We discovered that 46 of the phenotypes contained no such genes, illustrating that the addition of more variants did not introduce additional significant architecture.

### Genes and gene sets enriched for mutations in prioritized clusters

To test whether the shared significant genes in prioritized clusters fall into biologically relevant pathways, we performed gene-set enrichment analysis on each prioritized phenotype cluster reported by WINGS in our analysis of 81 UK Biobank phenotypes. We used the list of shared significant genes (*P*-value $<2.83\times 10^{-6}$, Bonferroni-corrected for 17,651 autosomal genes) for each prioritized cluster as input into Enrichr (Chen *et al.* 2013), and tested for enrichment of mutations in the Kyoto Encyclopedia of Genes and Genomes (KEGG) 2016 Pathway Database (Kanehisa *et al.* 2016). The full list of significantly enriched pathways is freely available in the PEGASUS-WINGS GitHub repository (see section *Data Availability*).

For each prioritized cluster in Figure 3 and Figure S11, we determined whether shared significant genes had at least one prior variant level association in the GWAS catalog (https://www.ebi.ac.uk/gwas/) (Table S2). Of the eight prioritized clusters identified in our analysis, seven contain shared significant genes that had been previously associated with at least one cluster phenotype. Phenotypes in the Metabolic cluster share two significant genes, and *NCR3* has been previously associated with both diabetes mellitus (Tomer *et al.* 2015) and IgG glycolysation (Lauc *et al.* 2013). Immunological cluster 1 contains three phenotypes that share 181 significant genes, 6 of which have been previously associated with one of the member phenotypes. Four of these have been previously associated with psoriasis: *C6orf10* (Lee *et al.* 2018), *HCP5* (Liu *et al.* 2008; Lee *et al.* 2018; Aterido *et al.* 2019), *MICA* (Lee *et al.* 2018), and *POUSF1* (Zhang *et al.* 2009). *HCP5* encodes a lncRNA in the MHC region whose hypomethylation has been associated with a CpG site with consequences for development of ankylosing spondylitits, another immunological disease (Coit *et al.* 2019). Two of the shared significant genes have been previously associated with celiac disease: *HLA-DQA1* (van Heel *et al.* 2007; Dubois *et al.* 2010) and *NOTCH4* (Östensson *et al.* 2013).

Immunological cluster 2 is made up of six phenotypes that share 96 significant genes, and 18 of these have been previously associated with at least one member phenotype. Three genes have been previously associated with more than one of the member phenotypes. *HLA-DQA1* has been previously associated with asthma (Dahlin *et al.* 2019), rheumatoid arthritis (RA; Jiang *et al.* 2015), and type 1 diabetes (Cooper *et al.* 2008). *HLA-DQB1* and *HLA-DRA* have both been previously been associated with asthma (Demenais *et al.* 2018; Shrine *et al.* 2019), but each has also been associated to another phenotype; *HLA-DQB1* to hypothyroidism (Pickrell *et al.* 2016) and *HLA-DQB1* to RA (Jiang *et al.* 2015). Moreover, 15 other genes have been associated with one member phenotype, and 10 of these (*BRD2*, *BTNL2*, *CDSN*, *CFB*, *HCP5*, *HLA-DOA*, *MICB*, *NOTCH4*, *PBX2*, *PSORS1C1*) have been associated with asthma across four studies (Hirota *et al.* 2011; Tomer *et al.* 2015; Almoguera *et al.* 2017; Demenais *et al.* 2018). Of the remaining five genes, three [*MICA* (Aterido *et al.* 2019), *NCR3*, and *TAP2* (Tomer *et al.* 2015)] have been previously associated with type 1 diabetes, while *APOM* (Hu *et al.* 2011) and *HLA-DRB5* (Jiang *et al.* 2014) have been associated with RA.

Using the KEGG pathway database and a list of the shared significant genes in Immunological cluster 2, an interesting pattern appears. For example, *HLA-DRA*, *HLA-DOA*, and *HLA-DQA1* are all components of pathways that play a role in hypothyroidism, RA, asthma, and antigen processing and presentation (Kanehisa *et al.* 2016). These three genes, thus, represent a shared core set of genes between phenotypes, and offer confirmation that WINGS has the power to detect a set of previously validated network interactions in a group of phenotypes. Asthma, in particular, has interesting genetic and environmental mechanisms in each of these three genes. In severe cases of asthma, epithelial gene expression of *HLA-DOA* has been shown to be significantly reduced in the central airways (Singhania *et al.* 2017). *HLA-DQA1*, in a study of individuals from both high and low socioeconomic status, was shown to be deferentially transcribed pointing to a gene by environment effect on the MHC region in disease development (Chen *et al.* 2009). Finally, *HLA-DRA* contains a nonsynonymous SNP in a coding region previously associated with asthma development in an independent dataset (Song and Lee 2013).

The Polyp cluster, whose member phenotypes are “polyp of colon” and “rectal polyp,” share 43 significant genes; however, none of them have been associated with either of those exact phenotypes. Interestingly, seven of the shared significant genes have been associated to colorectal cancers, including *UTP23* (Al-Tassan *et al.* 2015), *GREM1* (Whiffin *et al.* 2014), SCG5 (COGENT Study *et al.* 2008), *SMAD7* (Hofer *et al.* 2017), *CABLES2* (Schmit *et al.* 2018), *LAMA5* (Houlston *et al.* 2010), and *PREX1* (Lu *et al.* 2019). It is important to note the five phenotypes in the Assorted cluster share no shared significant genes.

The Alzheimer’s/Dementia cluster contains Alzheimer’s disease and vascular dementia, which share eight significant genes, all of which have been previously associated with one phenotype. *PALM2* was shown by Pottier *et al.* (2018) to be associated with frontotemporal dementia. The remaining seven genes, including *APOC1*, *APOC2*, *APOC4* (Marioni *et al.* 2018), *APOE* (Ramanan *et al.* 2014), *CLPTM1* (Jansen *et al.* 2019), *PVRL2* (Jun *et al.* 2017), and *TOMM40* (Cruchaga *et al.* 2011) have all been associated with Alzheimer’s disease. The mechanistic role of *APOE* in the pathogenesis of Alzheimer’s remains unclear, but is widely thought to interact in the metabolism and aggregation of amyloid-*β* in the brain (Kanekiyo *et al.* 2014).

The two phenotypes in the Kidney cluster share nine significant genes, and only *OVOL1* has been previously associated with a related phenotype (urate levels) by Köttgen *et al.* (2013). Lastly, member phenotypes of the liver cluster shared 25 significant genes, and three have been previously associated with nonalcoholic fatty liver disease. They include *GATAD2A* (Kawaguchi *et al.* 2018), *PNPLA3* (Kitamoto *et al.* 2013), and *SAMM50* (Chung *et al.* 2018).

### Previous methods and comparative dendrogram analysis

In this section, we present the results of multiple comparative dendrogram analyses to assess the performance of WINGS applied to genes with a ±50 kb boundary relative to other methods and gene score inputs. Table 4 shows the proportion of phenotype pairs with a significant cross-trait heritability estimate for each WINGS prioritized cluster shown in Figure 3. Significant cross-trait heritability estimates are indicative of two phenotypes having highly correlated variant level association statistics after correcting for inflation explained by LD, as defined in Bulik-Sullivan *et al.* (2015a). Multiple pairs of phenotypes are identified in both analyses; however, we note that the advantage of WINGS is its ability to identify clusters of phenotypes beyond pairs while simultaneously taking into account the pairwise distance among all phenotypes. We did not perform this analysis for the Alzheimer’s/Dementia cluster as Alzheimer’s returned no cross-trait heritability estimates with any other phenotype. Overall, we find that cross-trait LD score regression is not sufficient to identify clusters of phenotypes that are identified using the WINGS algorithm.

**Table 4:** Percentage of significant pairwise cross-trait heritability estimates within each WINGS prioritized cluster

Cluster	Cluster size	Phenotype pairs recovered(%)
Kidney cluster	2	100
Liver cluster	2	0
Polyp cluster	2	100
Metabolic cluster	8	46
Immunological 2 cluster	6	27
Assorted cluster	5	40
Immunological 1 cluster	3	33

**Figure 4:**
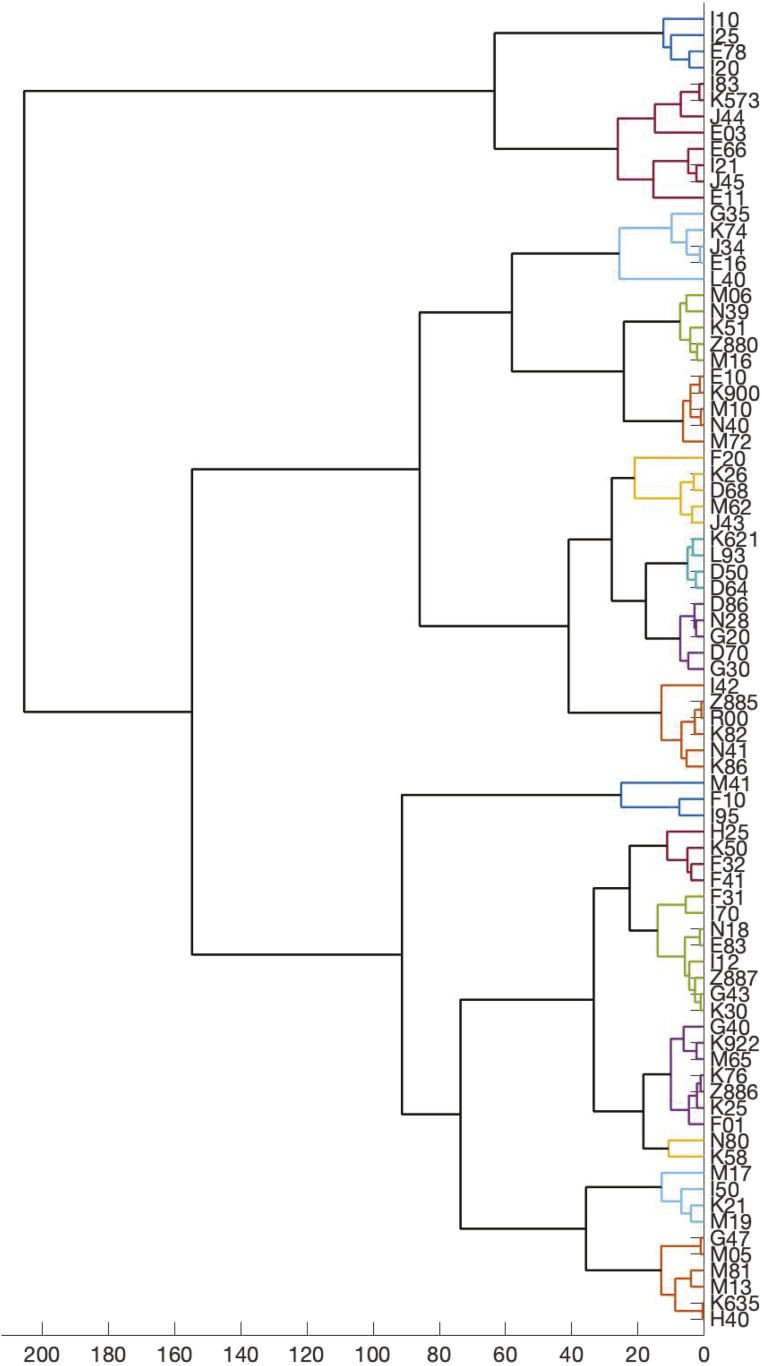
disPCA from 81 case–control phenotypes in the UK Biobank. We show the dendrogram resulting from applying Ward clustering to 81 case–control phenotypes based on their Euclidean distances in the space defined by the first two principal components of a minSNP gene score matrix. disPCA does not offer an intuition as to which clusters should be examined, and differentiation between internal branch lengths is less pronounced than when using the WINGS algorithm. The colored branches represent prioritized clusters identified by the WINGS algorithm. Note that all of the phenotypes are now within a prioritized cluster; we hypothesize that this is because disPCA reduces the gene score matrix to two dimensions so that all of the phenotypes lie within the same plane and phenotype-to-phenotype distances are condensed (see Figure S13).

To apply WINGS to the disPCA method developed by Chang and colleagues (Chang and Keinan 2014), we generated a tree for the 81 phenotypes analyzed in this study using their methodology. Figure 4 is the result of applying WINGS to phenotypes using pairwise distances among phenotypes defined by the first two PCs of a minSNP gene score matrix (see section *Comparison of WINGS to cross-trait LD score regression*, *disPCA*, *and other regional trees*). The resulting tree lacks an internal branch length structure that would differentiate clusters from one another. Moreover, all of the phenotypes lie within a prioritized cluster in this framework so that now no single collection of phenotypes seems to bear any significant shared genetic architecture. We hypothesize that this behavior is due to the fact that disPCA reduces the gene score matrix to two dimensions so that all of the phenotypes lie on the same plane, and phenotype-to-phenotype distances are condensed. The positions of each point within the space are displayed in Figure S13, which further demonstrates how the disPCA analysis does not result in clearly defined clusters and that the phenotype clusters identified by WINGS are not easily differentiable in the PC space.

In addition to only including genes and their ±50 kb regions as features, we also computed PEGASUS scores for intergenic regions, and observe that the topology of the tree is similar [dissimilarity index from Morlini and Zani (2012) between Figure 3 and Figure S14 is Z = 0.1091]. Second, we performed an analysis using imputed genotype data provided by the UK Biobank. We included all of the genotype variants that were in the original analysis as well as imputed variants passing thresholds described in *Comparison of WINGS to cross-trait LD score regression*, *disPCA*, *and other regional trees*. The resulting dendrogram and branch length distribution are shown in Figures S16 and S17, respectively. While slightly more dissimilar than the intergenic tree (Z = 0.1379), the topology of the resulting dendrogram shows the preservation of prioritized clusters or subsets of prioritized clusters. By comparison, when the buffer region around genes is set to zero, the dissimilarity index, Z = 0.1240, is marginally lower. The resulting dendrogram and branch length distribution are illustrated in Figures S18 and S19. Finally, when analyzing the genome where regional association statistics (*i.e.*, gene scores) are calculated for each of the 33,686 independent haplotype block, the topology of the tree is highly conserved when compared with our primary result (Z = 0.1276). The resulting dendrogram and branch length distributions are shown in Figure S20 and Figure S21. In each of these analyses, we find that there is no substantial change in the dendrogram and therefore the genotyped common variants can adequately detect shared significant genetic architectures.

### Parameter sensitivity analysis

As noted in the section *WINGS*, *a new method for automatic phenotype cluster detection and ranking*, there are two parameter choices in WINGS: the cluster size threshold and the outlier criterion. In the software for WINGS, these parameters are optionally user-defined, where the default cluster size threshold is conservatively set to $\frac{N}{3}$ (meaning the size of any prioritized cluster will not be more than approximately one-third of the total number of phenotypes in the input data), and the default outlier criterion is based on median absolute deviations method. Here, we test the robustness of WINGS with respect to these two parameters and analyze the results to gain insight into how different parameter choices will impact how WINGS identifies the prioritized clusters.

For this analysis, we apply WINGS to a single simulated gene score matrix that contains 100 phenotypes and has 75% shared genetic architecture. That is, 75% of the 175 significant genes in each phenotype are shared across all phenotypes within a cluster. There are 13 clusters in this simulation, ranging in size from two to seven phenotypes per cluster. We apply WINGS to this gene score matrix while varying the cluster size threshold from 2 to 19 in single increments. For each cluster size threshold, we run WINGS using the median absolute deviations outlier criterion (herein referred to as the median outlier criterion), mean outlier criterion, quartile outlier criterion, and Grubbs’ outlier criterion.

With the median outlier criterion, branch length gaps that are more than three scaled median absolute deviations away from the median are considered an outlier (Rousseeuw and Croux 1993; Huber 2011; Leys *et al.* 2013; MATLAB Data Import and Analysis 2018). In contrast, branch length gaps that are more than three SD from the mean are considered outliers under the mean outlier criterion (Leys *et al.* 2013; MATLAB Data Import and Analysis 2018). Under the quartile outlier criterion, branch length gaps that are >1.5 interquartile ranges above the upper quartile, or below the lower quartile, are marked as outliers (Rousseeuw and Croux 1993; Huber 2011; Leys *et al.* 2013; MATLAB Data Import and Analysis 2018). Finally, the Grubbs’ outlier criterion uses Grubbs’ test to remove outliers one-by-one based on statistically testing the hypothesis that the data contain no outliers (Grubbs 1950; MATLAB Data Import and Analysis 2018). The appropriate choice of outlier criterion depends on the input data and objectives. For example, the mean outlier criterion is faster but less robust than the median outlier criterion (Rousseeuw and Croux 1993; Leys *et al.* 2013; MATLAB Data Import and Analysis 2018). The mean and Grubbs’ methods assume the input data are normally distributed, whereas the quartile and median methods are useful when the input data are assumed not to be normally distributed (Grubbs 1950; Rousseeuw and Croux 1993; Huber 2011; Leys *et al.* 2013; MATLAB Data Import and Analysis 2018).

**Figure 5:**
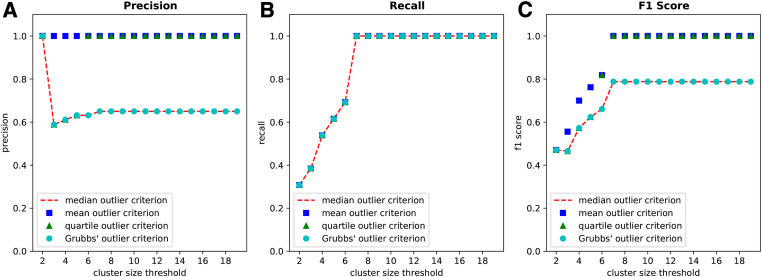
Performance of WINGS on a simulated gene score matrix with shared genetic architecture across parameter regimes. We apply WINGS to a single simulated gene score matrix containing 100 phenotypes with 75% shared genetic architecture (meaning, 75% of the 175 significant genes in each phenotype are shared across all phenotypes within a cluster). There are 13 clusters in this simulation, ranging in size from two to seven phenotypes per cluster. We show the performance of WINGS in terms of precision (A), recall (B), and F1 score (C) for an increasing sequence from cluster size thresholds (2–19) and for each outlier criterion method (median, mean, quartile, Grubbs’). Precision is the percentage of prioritized clusters (identified via WINGS) that are ground truth clusters. Recall is defined as the percentage of ground truth clusters that are also prioritized by WINGS. F1 score is twice the product of precision and recall divided by the sum of precision and recall.

The precision, recall, and F1 scores of this parameter test are presented in Figure 5. Precision is the percentage of prioritized clusters (identified via WINGS) that are ground truth clusters; recall is defined as the percentage of ground truth clusters that are also prioritized by WINGS; the F1 score is twice the product of precision and recall divided by the sum of precision and recall.

As shown in Figure 5B, the recall rate of WINGS is 100% when the cluster size threshold is greater than six, regardless of the choice of outlier criterion. This means that all of the ground truth clusters are correctly prioritized by WINGS for each choice of outlier criterion, as long as the cluster size threshold is seven or higher. As noted above, the simulated data in this experiment has ground truth clusters containing anywhere from two to seven phenotypes, and therefore the cluster size threshold must be at least seven in order for WINGS to consider all of the ground truth clusters as candidates for the prioritized clusters.

In Figure 5, A and C, we observe that the mean and quartile outlier methods perform better than the median and Grubbs’ outlier methods in terms of precision rate and F1 score. In particular, the precision rate of WINGS is 100% when using the mean outlier method, for all cluster size thresholds; said another way, when using the mean outlier method with any choice of cluster size threshold, all of the prioritized clusters identified by WINGS are indeed ground truth clusters. Similarly, the precision rate of WINGS is 100% when using the quartile outlier method, as long as the cluster size threshold is greater than five. By combining these results with the recall rates, WINGS yields an F1 score of 100% using either the mean or quartile outlier method, as long as the cluster size threshold is at least seven (Figure 5C). In contrast, the precision rate of WINGS is 65% when using either the median or Grubbs’ outlier method with a cluster size threshold greater than six (Figure 5A). We note that WINGS prioritizes seven false positive clusters, along with the 13 ground truth clusters, when using either the median or Grubbs’ outlier method with a cluster size threshold greater than six. Consequently, the resulting the F1 scores of WINGS in these parameter regimes is 78.79% (Figure 5C).

Overall, these results demonstrate the robustness of WINGS with respect to the cluster size threshold parameter across all performance metrics, and the robustness of WINGS with respect to the outlier criterion in terms of its recall rates. This experiment further reveals that WINGS may be more likely to identify false positive prioritized clusters when using the median or Grubbs’ outlier criterion. Nevertheless, we use the median absolute deviation method in our experiments since it has been observed as one of the more robust outlier detection methods and does not assume the input data are normal (Rousseeuw and Croux 1993; Huber 2011; Leys *et al.* 2013; MATLAB Data Import and Analysis 2018). Because our objective is to identify new groups of phenotypes with shared genetic architecture, we prefer to identify false positive clusters rather than to risk false negative clusters. Moreover, by further testing to see which prioritized clusters contain shared significant genes that fall into biologically relevant pathways, we could filter out any possible false negative clusters in the empirical data. Finally, we highlight that WINGS ranks the resulting phenotype clusters based on their level of significance so we have an additional measure of confidence associated to each of our prioritized clusters across all parameter choices.

## Discussion

Although biobank-scale datasets—in which multiple phenotypes are assayed and/or surveyed in tens of thousands to hundreds of thousands individuals—are becoming increasingly available to medical genomics researchers, approaches for leveraging these datasets to identify shared architecture among phenotypes are still in their infancy. Existing approaches for analyzing the shared genomic underpinnings of multiple phenotypes focus on colocalizing variant-level signals (Denny *et al.* 2016; Pickrell *et al.* 2016). However, recent approaches that aggregate SNP-level genotype-phenotype association statistics within genes into a gene-level association score have gained power to detect biologically relevant and interpretable genes and pathways enriched for mutations in complex diseases (Carbonetto and Stephens 2013; Lamparter *et al.* 2016; Nakka *et al.* 2016; Wang *et al.* 2017; Zhu and Stephens 2018), and offer insight into the roles of genetic heterogeneity and interactions among variants in generating complex diseases.

Here, we present a new method, Ward clustering to identify Internal Node branch length outliers using Gene Scores (WINGS), for identifying phenotypes that share significant genetic architecture based on germline genetic data matched with binary or quantitative phenotypes from mega-biobanks. WINGS leverages Ward hierarchical clustering applied to gene-level association scores for the phenotypes of interest, and goes beyond past clustering applications to GWA studies of multiple phenotypes [*e.g.*, disPCA (Chang and Keinan 2014) and cross-train LD score regression (Bulik-Sullivan *et al.* 2015b)] by (*i*) its ability to simultaneously analyze and cluster all phenotypes rather than study pairwise correlations (see section *Previous methods and comparative dendrogram analysis*, Table 4) and (*ii*) providing a thresholding algorithm for identifying and ranking prioritized clusters of phenotypes. WINGS offers an innovative way to detect signals of statistical pleiotropy by identifying genes that are associated with complex phenotypes and presenting them in an easily interpretable way. We note that the thresholding step in WINGS offers a useful visualization for interpreting results: while dendrograms depict the hierarchical architecture of clusters (Figures 3 and Figure S8), the sorted branch lengths WINGS provides as output are intuitive to read, demonstrate a clear ranking of clusters, and identify prioritized clusters (Figures S9 and S11).

In this study, we apply WINGS to simulations and data from the UK Biobank and show that it is sensitive to identifying phenotype clusters characterized by enrichment of mutations in a “core set” of genes across cases; future applications of WINGS could also incorporate regulatory and intergenic association signals into analysis (see also Figures S14 and S15), or focus solely on enrichment of association signals in untranslated genomic regions. Given concerns over whether GWA data contain signals of genetic architecture, we note that our simulations indicate that WINGS is sensitive to shared significant genes (that is, genes enriched for phenotype-associated mutations, identified by applying WINGS to –log_10_ transformed gene-level association *P*-values).

Figure 3 and Figure S8 and S9 suggest that WINGS can offer insight into shared genetic architecture underlying comorbid phenotypes, as well as phenotypes that may often be misdiagnosed for one another, such as vascular dementia and Alzheimer’s disease (Bozeat *et al.* 2000; Perrin *et al.* 2009). As validation of the phenotype clusters identified in our analyses of the UK Biobank, Table S2 shows that genes significantly associated with phenotypes in our analyses have been previously associated via variant-level GWA studies with some, but not all, phenotypes in clusters in Figure 3 and Figure S11.

Clustering high-dimensional features will always be relative to the input data. We ran several comparative dendrogram analyses to assess the performance of WINGS applied to other types of gene score inputs, including PCs from the disPCA method (Chang and Keinan 2014), PEGASUS scores with intergenic regions, imputed genotype data, genes with no buffer region, and independent haplotype blocks. Overall, we found that disPCA features do not result in clearly defined clusters, while the other variations of the gene score matrix do not vary substantially from the original feature inputs (gene scores with ±50 kb regions) (see section *Previous methods and comparative dendrogram analysis*; Figure 4 and Figure S13–S21). Moreover, we underscore that our analysis of 26 phenotypes in the UK Biobank [chosen based on having been studied by both Pickrell *et al.* (2016) and Shi *et al.* (2016), as well as having over 100 cases in the UK Biobank] also recovers multiple prioritized clusters of phenotypes identified in our full set of 81 phenotypes: Alzheimer’s/Dementia, Metabolic, and Immunological 2 (Figure S8).

While our objective and approach differ significantly from the studies of Pickrell *et al.* (2016) and Shi *et al.* (2016) [we focus on case-control studies and they analyzed many quantitative phenotypes; Pickrell *et al.* (2016) test for variant-level pleiotropy among 42 complex traits, whereas Shi *et al.* (2016) study the contribution of genomic regions to narrow-sense heritability of 30 complex traits], we observe similarities across our results and their published studies. In comparing the prioritized clusters from WINGS applied to the 26 binary chronic illness phenotypes from the UK Biobank and the results presented in Shi *et al.* (2016), we observe that both studies find strong connections between RA and ulcerative colitis (UC). Shi *et al.* 2016 show in their Figure 8 that RA and UC, in particular, have a high fraction of total SNP heritability, among other immunologically relevant phenotypes, along the HLA region (see Figures S8 and S9 to observe that RA and UC reside in the same prioritized cluster when WINGS is applied to the 26 binary chronic illness phenotypes from the UK Biobank on the −log_10_ scale). In Pickrell *et al.* (2016), overlapping SNP-level association signals identify clusters of related traits and these clusters have nontrivial overlap with the prioritized clusters identified by WINGS. Notably, type 2 diabetes and lipid traits cluster together in both the analysis of overlapping association signals in Pickrell *et al.* (2016) and in the prioritized clusters when WINGS is applied to the 26 phenotype dataset on the –log_10_ scale (see Figures S8 and S9). Moreover, RA and UC show patterns of high overlap in Figure 2 of Pickrell *et al.* (2016), and these phenotypes similarly reside in the same prioritized clusters when WINGS is applied to the 26 binary chronic illness phenotypes from the UK Biobank on the −log_10_ scale (see Figures S8 and S9).

Next, we offer some caveats for future applications of WINGS and potential future directions for the development of methods to identify shared genetic architecture among multiple phenotypes in mega-biobanks. First, our goal here was to validate WINGS with simulations and to generate hypotheses regarding shared genetic architecture among complex phenotypes in the UK Biobank. We did not seek to replicate our results from applying WINGS to data, an increasingly common challenge for mega-biobank analyses (Huffman 2018). However, our validation with simulations and annotation of previously identified genes reinforces that we are reliably detecting shared genetic architecture (see File S1 for an extensive list of replication citations). Second, based on Figure S1, clustering does not work well when applied to a group of phenotypes that have different genetic architectures, in particular quantitative phenotypes and binary phenotypes, as the differences in gene score distribution will cluster binary and continuous in a noninformative manner. One approach that could help overcome this challenge is the development of a gene score that incorporates both effect sizes and their SE into calculation (Stephens 2016), but this is outside the focus of this study.

Third, although WINGS is robust to the cluster size threshold and outlier criterion (see section *Parameter sensitivity analysis*; Figure 5), WINGS is more sensitive to the clustering criterion and the gene scores used as input. We focused on Ward hierarchical clustering here, due partly to its performance on simulated phenotype clusters (Table 2), and to its assumption that clusters are round; because clusters are hard to find in a high-dimensional space, this may be a conservative choice. In Section S3, we applied our method using other clustering criteria (single linkage, average linkage, and complete linkage clustering) to the 81 phenotypes we analyzed from the UK Biobank, and compared the resulting prioritized clusters. We chose PEGASUS gene-level *P*-values as input to WINGS due to (*i*) our previous exploration of the power of PEGASUS (Nakka *et al.* 2016); which demonstrated that PEGASUS is not biased by gene length, and computes more precise *P*-values than VEGAS (Liu *et al.* 2010) and SKAT (Wu *et al.* 2011); and (*ii*) because the model of correlated SNP-level *P*-values underlying PEGASUS is the same as that of another gene-level association method PASCAL (Table S3).

Future applications and extensions of WINGS may focus on a number of questions regarding shared genetic architecture among phenotypes. For example, Pickrell *et al.* (2016) tested variants for true pleiotropy, while our current implementation of WINGS cannot differentiate between phenotypic relationships defined by clinical comorbidity *vs.* causal dependence (see also Denny *et al.* 2016). We also assume that ICD10 codes are reliable indicators of disease status, which may not be the case (Shivade *et al.* 2014; Hripcsak *et al.* 2018); for example, ICD10 codes may reflect billing codes for tests but not validated diagnoses. One potential follow-up analysis is to experimentally test the pleiotropic effect of shared significant genes in prioritized clusters, as these would be ideal candidates for drug design. WINGS provides a new hypothesis generating framework that can be used to identify primary candidate genes for pleiotropic effects contributing to multiple human diseases. As new biobank data includes whole genome or exome data, reanalysis could provide further insight into how rare variants affect potentially pleiotropic genes. As natural language processing is brought to bear on electronic medical records, and biobanks merge quantitative test results with germline genetic data [a few example studies from the UK Biobank are Jani *et al.* (2019) and Havdahl *et al.* (2019)], algorithms such as WINGS could be used to identify genetic associations for multiple biomarkers and/or comorbidities. WINGS is sensitive to identifying shared mutated genes from −log_10_-transformed gene scores, and we interpret the genes underlying prioritized clusters in the output of WINGS as core genes underlying the clustered phenotypes (Boyle *et al.* 2017). Integrating results from WINGS with tissue-specific expression data would further test this hypothesis; similarly, WINGS could further be applied to study protein interaction networks using, for example, the topological scores of Sardiu *et al.* (2019) as inputs for the identification of prioritized subnetworks. Lastly, WINGS could also be extended to test for differential genetic architecture among ancestries (Martin *et al.* 2017)—a fundamental question to which mega-biobanks can offer unique insights in the coming years.
